# Exposure to statins and risk of common cancers: a series of nested case-control studies

**DOI:** 10.1186/1471-2407-11-409

**Published:** 2011-09-26

**Authors:** Yana Vinogradova, Carol Coupland, Julia Hippisley-Cox

**Affiliations:** 1 Division of Primary Care, 13th floor, Tower Building, University Park, Nottingham, NG2 7RD, UK

## Abstract

**1 Abstract:**

**Background**

Many studies and meta-analyses have investigated the effects of statins on cancer incidence but without showing consistent effects.

**Methods**

A series of nested case-control studies was conducted covering 574 UK general practices within the QResearch database. Cases were patients with primary cancers diagnosed between 1998 and 2008. The associations between statin use and risk of ten site-specific cancers were estimated with conditional logistic regression adjusted for co-morbidities, smoking status, socio-economic status, and use of non-steroidal anti-inflammatory drugs, cyclo-oxygenase-2 inhibitors and aspirin.

**Results**

88125 cases and 362254 matched controls were analysed. The adjusted odds ratio for any statin use and cancer at any site were 1.01 (95%CI 0.99 to 1.04). For haematological malignancies there was a significant reduced risk associated with any statin use (odds ratio 0.78, 95%CI 0.71 to 0.86). Prolonged (more than 4 years) use of statins was associated with a significantly increased risk of colorectal cancer (odds ratio 1.23, 95%CI 1.10 to 1.38), bladder cancer (odds ratio 1.29, 95%CI 1.08 to 1.54) and lung cancer (odds ratio 1.18, 95%CI 1.05 to 1.34). There were no significant associations with any other cancers.

**Conclusion**

In this large population-based case-control study, prolonged use of statins was not associated with an increased risk of cancer at any of the most common sites except for colorectal cancer, bladder cancer and lung cancer, while there was a reduced risk of haematological malignancies.

## 2 Background

Multiple randomised controlled trials have demonstrated the benefits of statins in improving survival for patients with ischaemic heart disease [1-5] and this has caused a substantial increase in statin use. While there are definite benefits from statins in reduction of mortality in high risk patients, uncertainties remain about whether statins might increase or decrease the risk of cancer[6-8]. This is important because statins are prescribed for extended periods to large numbers of patients.

The effect of long-term statin use is quite complex because the multiple properties of statins go beyond lipid lowering. There is evidence that statins increase endothelial dysfunction [9] and lower inflammatory markers[10] but it is still not clear whether they may affect the risk of cancer. Experimental data (primarily using rats) have shown both carcinogenicity of statins[11] and no effect on carcinogenesis[12]. Some studies performed on human cancer cells *in vitro *have suggested that statins may be chemo-prophylactic against various types of cancer including colon[13] and breast cancer[14,15]. It has also been found that statins may suppress the growth of cancer cells in vitro by causing the cells to pause in the G1 phase of the mitotic cycle[16] and by increasing cell death[17].

There have been many randomised controlled trials of statins, but cancer has never been a primary outcome. The numbers of cancer cases have been relatively small and the duration of the trials too short to detect the effect of statins on cancer risk. The results from 35 randomised control trials have been summarised in a meta-analysis[18] reporting no association between statin use and overall cancer risk. However, the latest published results of another randomised controlled trial, not included in the meta-analysis, on the use of a combination of simvastatin and ezetimibe in patients with aortic stenosis demonstrated an increased risk for any cancer (105 vs.70, P = 0.01)[19].

A number of observational studies were designed to assess risk of particular cancers in statin users and the results have been aggregated in a meta-analysis[7]. However, only some of the studies reported statin use of more than 5 years[20]. None of those findings were statistically significant except for one study reporting a decreased risk of prostate cancer, but based only on 42 statin users[21]. A recent study of statin use and ten common cancers[22] found a significantly reduced risk of haematological malignancies and an increased risk of endometrial cancer associated with more than 5 years of statin use.

All studies were smaller than the proposed one, and they were too dissimilar in their definitions of statin use to be analysed together: they either studied different types of statin or statin types were not specified. They also had differing lengths of intervention or follow-up, and included different confounding factors in their analyses.

Given the uncertainty regarding risk of cancer in association with statin usage, we designed a study to determine the risk for the most common incident cancers associated with taking statins including for prolonged periods using a very large population-based research database QRESEARCH. The size of the study has enabled us to adjust for use of other drugs and many potential confounding factors.

## 3 Methods

### 3.1 Study design, data source and population

We conducted a series of nested case control studies within a cohort of patients registered with practices in the UK contributing to the QRESEARCH database (version 20). The QResearch database (http://www.qresearch.org) is one of the largest general practice databases containing anonymised clinical records for over 11 million patients registered with 574 UK general practices. The information recorded on the database includes patient demographics (year of birth, sex, socio-demographic data derived from UK census 2001), characteristics (height, weight, smoking status), clinical diagnoses, symptoms, and prescribed medications including repeat prescriptions. The database has been validated by comparing birth rates, death rates, consultation rates, prevalence and mortality rates with other data sources, including the General Household Survey and the General Practice Research Database, and has demonstrated good levels of completeness and consistency[23,24]. Practices were included in the analysis only if they had complete data transmission until at least 1^st ^July 2008.

We identified an open cohort of patients registered with the study practices during the 10 year study period between 1st Jan 1998 and 1st July 2008. We then used READ codes to select all cases aged between 30 and 100 years with a first record of any cancer in the patients' electronic records occurring during the study period. Each case was linked to 5 controls alive and registered with the practice at the time of diagnosis of the case and matched by age, sex, practice and calendar time. Controls were allocated an index date which was the date on which their matched case was first diagnosed with cancer.

### 3.2 Exclusions

Cases with secondary cancers (READ codes: B56, B57, B58) were excluded. Cases and controls with a diagnosis of any cancer before the index date were excluded. In addition, for breast cancer, we excluded cases and controls with any prior record of mastectomy or prescriptions for tamoxifen since they could be breast cancer cases without a recorded diagnosis in their record. To ensure completeness of exposure data we also excluded temporary residents and patients with fewer than 6 years of medical records before the index date for the main analysis - and fewer than 10 years for the further analysis.

### 3.3 Primary outcomes

We determined the risks for the most common cancers in the UK[25], comparing these for patients prescribed statins against those not prescribed the drugs. The investigated cancers and corresponding READ codes were: Breast cancer (women, B34), Prostate cancer (men, B46), Lung cancer (B22), Bladder cancer (B49), Haematological malignancies (B6), Gastric cancer (B11), Oesophageal cancer (B10), Colorectal cancer (B13, B14), Pancreatic cancer (B17) and Melanoma (B32). As haematological malignancies cover a range of diseases, possibly differentially affected by statins, we also investigated leukaemia (B63-B6z), lymphoma (B60-B62) and myeloma (B63) separately.

### 3.4 Exposure variables

Statin exposure was determined based on all prescriptions for statins until 1 year before the index date (date of diagnosis or equivalent date for controls). The drugs included were atorvastatin, pravastatin, fluvastatin, cerivastatin, rosuvastatin, and simvastatin. Prescriptions in the year before the index date were ignored because including these could lead to results being affected by reverse causality - prescribing in cases in this period might be the result of consultations relating to early cancer symptoms before the recorded diagnosis and this could attenuate any protective effect or exaggerate any harmful effect.

For the main analysis, we considered a 60-month period comprising statin prescriptions for the 13 to 72 months prior to the index date. For the additional analysis, covering a follow-up of 10 years, the period considered was 98 months - for the 13 to 120 months prior to the index date.

Statin use was categorised in a number of ways. We considered a patient as a statin user if they had at least 2 prescriptions in the 60-month period (or the 98-month period for the 10-year analysis). We estimated the cumulative use of statins by extracting the duration of use for every prescription and, for groups of prescriptions with inter-prescription gaps of less than 60 days, we calculated overall course times from the start of the first prescription to the end of the last prescription. We then calculated cumulative use as the sum of all overall course times and for the main analysis categorized cumulative use for each patient as: no use; less than 12 months; 13 to 24 months; 25 to 36 months; 37 to 48 months; 49 to 60 months. A test for trend was performed using the actual number of months of use. For the further analysis, covering a follow-up period of 10 years, the categorisation of the time period for statin use was: no use; less than 12 months, 13 to 24 months, 25 to 48 months, 49 to 72 months, and more than 73 months.

If there were at least 2 prescriptions in the 60-month main study period (or in the 98-month additional study period), we conducted analyses for the following individual statin types: simvastatin, atorvastatin, cerivastatin, fluvastatin, pravastatin and rosuvastatin. For the most common types - simvastatin, atorvastatin and pravastatin - we also examined the effect of cumulative use on cancer risk.

Statin dosage was calculated as median dose across the observation time period, and was categorised as low, medium or high according to statin efficacy[26]. The effect of stopping statin usage on risk of cancer was investigated in the main study only by comparing the last prescription date in the study period with the date one year before the index date and categorising as: no statin use in the 13 to 72 months prior to the index date; still on statins; stopped statins 13 to 24 months before the index date; and stopped statins 25 or more months prior to the index date.

### 3.5 Potential confounding variables

We adjusted for variables which are established cancer risk factors: diabetes[27], rheumatoid arthritis[28], hypertension[29] and body mass index (< 25, 25-29.99, ≥ 30 kg/m^2^)[30], if recorded at least 1 year before the index date, and for smoking status (non-smoker, ex-smoker, current smoker) and individual Townsend deprivation score (measure of socio-economic status, in fifths), if recorded before the index date. The Townsend score was based on 2001 postcode-related census data, with higher scores indicating greater level of material deprivation and was used because there is a link between deprivation and incidence of some types of cancer[31]. We adjusted for cardiovascular disease as the main reason for statin therapy. For breast cancer we also accounted for any previous benign breast disease (fibrocystic disease, intraductal papilloma, fibroadenoma) and for family history of breast cancer. For colorectal cancer, additional confounders considered were colitis and Crohn's disease.

We also adjusted for use of traditional non steroidal anti-inflammatory drugs, cyclooxygenase-2 inhibitors and aspirin, as several studies have found protective effects for non-steroidal anti-inflammatory drugs and aspirin on various types of cancer[32,33], in particular on colorectal cancer[33]. We categorised the number of prescriptions for these drugs in the 60-month main study period as: none; 1 to 12; 13 to 24; and 25 or more (adding 25 to 48 and 49 or more for the 98-month additional study period); and adjusted for those categories in assessing cancer risk. We also included in the analyses use of other medications likely to increase the risk of cancer (hormone replacement therapy and oral contraceptive use for breast cancer analysis[34]) if there were at least 2 prescriptions of a drug in the 60-month main study period or 98-month additional study period.

### 3.6 Statistical analysis

We used conditional logistic regression to estimate odds ratios with 95% confidence intervals for cancer overall and each of the specific cancer sites. As body mass index, smoking status and Townsend deprivation score may be important confounders and have a certain amount of missing data, we used multiple imputation for the missing values[35,36]. We used the ICE procedure in STATA to obtain 5 imputed datasets and applied Rubin's rules to combine effect estimates and estimate standard errors to allow for uncertainty caused by the missing data. We repeated the imputation procedure for each type of cancer separately.

The initial analysis model determined the unadjusted odds ratios for each cancer associated with statin prescriptions according to: any use of statins in the 60-month study period (at least 2 prescriptions in the 13 to 72 months before the index date); cumulative duration of use; and the median prescribed dose. A multivariate model determined the odds ratio for each cancer associated with statin prescriptions adjusted for the potential confounding effects of variables listed above. For comparison with the analyses using imputed data for smoking status and body mass index, we also ran complete case analyses including only cases and controls with complete data as well as analyses using indicator variables for missing categories of smoking, deprivation and body mass index.

We used all the available data on the QResearch database so did not do a pre-study sample size calculation. According to post-hoc calculation, in order to detect an odds ratio of 0.8 (or 1.2) with 80% power at 1% significance for an exposure that occurs in 15% of controls a sample of 2685 cases (or 3424 cases) would be needed. We checked that we had sufficient power for analysis of the six commoner cancers. STATA v 10 was used for all the analyses. We used a 1% significance level to account for the multiple outcomes.

## 4 Results

Overall there were 118,780 patients with a recorded diagnosis of cancer at any site within the study period. 3,810 patients had diagnoses of secondary cancers so were removed from the analysis. Thirty six patients were coded with cancers applicable only to the other gender and were also removed. For breast cancer 370 cases and 302 controls with a previous history of mastectomy were excluded as were a further 685 cases and 471 controls with a previous history of tamoxifen use. This left a total of 113,879 patients with a first diagnosis of cancer during the study period and 568,958 controls. After removing patients with less than 6 years of medical records there were 88,125 cases of primary cancer matched with 362,254 controls. Eighty-one percent of cases and 71% of controls also had complete data for 10 years of follow-up. The proportions of cases with different types of cancer were similar to proportions in cancer registration statistics in England for 2003[37] for patients older than 30 years.

### 4.1 Baseline characteristics

Table 1 shows baseline characteristics for cases of cancer at any site and their matched controls. Fifty-three percent of the cases were men; the median age at diagnosis was 69 years (interquartile range: 60 to 77). Seventy six percent of cases and 73% of controls had complete data for body mass index, smoking status and Townsend deprivation score.

**Table 1:** Baseline characteristics for all cases with primary cancer and their matched controls with at least 6 years of medical records

	Cases (N = 88125)	Controls (N = 362254)
**Sex**		

female	41749 (47.4)	170173 (47.0)

male	46376 (52.6)	192081 (53.0)

**Age band (years)**		

30-54	13151 (14.9)	49906 (13.8)

55-64	19638 (22.3)	80107 (22.1)

65-74	26758 (30.4)	111698 (30.8)

75-84	25013 (28.4)	106278 (29.3)

85 +	3565 (4.0)	14265 (3.9)

**Deprivation**		

Townsend quintile 1, most affluent	22072 (25.0)	92287 (25.5)

Townsend quintile 2	18998 (21.6)	79067 (21.8)

Townsend quintile 3	17338 (19.7)	71358 (19.7)

Townsend quintile 4	15325 (17.4)	61767 (17.1)

Townsend quintile 5, most deprived	11896 (13.5)	45971 (12.7)

Townsend missing	2496 (2.8)	11804 (3.3)

**Body mass index (kg/m**^**2**^**)**		

15-24	26721 (30.3)	105883 (29.2)

25-29	27285 (31.0)	108803 (30.0)

30-49	12922 (14.7)	51413 (14.2)

not recorded	21197 (24.1)	96155 (26.5)

**Smoking status**		

non-smoker	54307 (61.6)	233135 (64.4)

ex-smoker	7567 (8.6)	23842 (6.6)

current smoker	17275 (19.6)	54869 (15.1)

not recorded	8976 (10.2)	50408 (13.9)

**Co-morbidities**		

Cardiovascular disease	14278 (16.2)	58123 (16.0)

Diabetes	7115 (8.1)	26802 (7.4)

Hypertension	27104 (30.8)	109797 (30.3)

Osteoarthritis	12807 (14.5)	52586 (14.5)

Rheumatoid arthritis	1310 (1.5)	5132 (1.4)

Colitis^1^	124 (1.1)	293 (0.6)

Crohn's disease^1^	28 (0.2)	109 (0.2)

Benign breast disease^2^	1094 (7.0)	2937 (4.7)

Family history of breast cancer^2^	539 (3.4)	1249 (2.0)

**Medications (in previous 13-72 months)**		

NSAID	35697 (40.5)	140642 (38.8)

COX2 inhibitors	6901 (7.8)	26974 (7.4)

Aspirin	19895 (22.6)	79067 (21.8)

Hormone replace therapy^2^	3289 (21.0)	10973 (17.4)

Oral contraceptive pill^2^	523 (3.3)	1638 (2.6)

^1) ^Based only on cases with colorectal cancer and their controls

^2) ^Based only on female cases with breast cancer and their controls

Cases and controls had similar patterns of co-morbidity except for diabetes (8.1% in cases vs. 7.4% in controls). The difference in proportion of diabetic patients was most marked in pancreatic cancer cases (12.7% vs. 8.3% in controls).

### 4.2 Statin exposure

Overall 15.5% of cases and 15.1% of controls had at least 2 statin prescriptions between 13 to 72 months prior to the index date. Most of the statin users (95% of cases and controls) had statin prescriptions for more than a year. Median numbers of scripts for statin users were 19 (interquartile ranges, 9 to 32) for cases and for controls. Median numbers of months on statin were 28 for cases and controls (interquartile ranges, 12 to 50 for cases and 12 to 49 for controls).

The most frequently prescribed statins were simvastatin (9.2% of cases and 9.0% of controls), atorvastatin (6.1% of cases and 5.9% of controls) and pravastatin (1.6% of cases and controls). The other statins were prescribed to less than 1% of the population. Very few atorvastatin users had low dose prescriptions (3 cases and 28 controls) and few pravastatin users had high dose prescriptions (4 cases and 12 controls). Simvastatin dosage was distributed evenly. Long-term statin use was associated with higher dose: in patients prescribed statins for more than 4 years, 42% of cases and 43% controls were on high doses compared with 31% cases and 31% controls on high doses in patients prescribed statins for less than 4 years.

**Figure 1:**
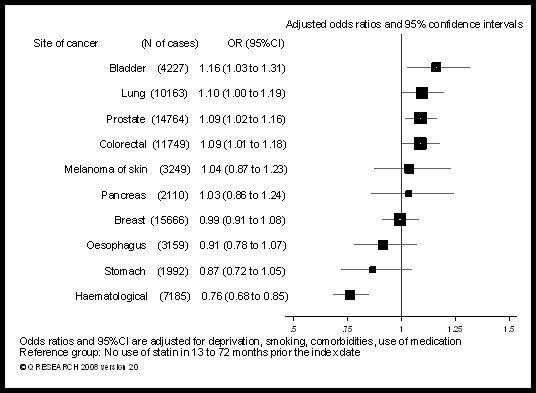
**Risk of cancer in patients using statins for more than 365 days in 13 to 72 months prior to the index date**.

The results of the main analyses, based on patients with at least 6 years of medical records, are shown in Tables 2, 3, 4 and Figure 1. Table 5 shows the odds ratios for each cancer according to cumulative duration of statin use in patients with at least 10 years of medical records.

**Table 2:** Use of statins in cases and controls in 13 to 72 months prior the index date by cancer site (in cases and matched controls with at least 6 years of medical records)

Cancer	Total number of cases	Total number of controls	N of statin users in cases (%)	N of statin users in controls (%)	Unadjusted OR (95%CI)		**Adjusted**^# ^**OR (95%CI)**		P-value
**breast**†	15666	62938	1481 (9.5)	6227 (9.9)	0.98	(0.92 to 1.04)	1.00	(0.93 to 1.08)	0.993

**prostate**	14764	61853	2774 (18.8)	11508 (18.6)	1.03	(0.98 to 1.08)	1.08	(1.01 to 1.14)	0.016

**colorectal**‡	11749	48624	2000 (17.0)	7770 (16.0)	1.12	(1.06 to 1.19)	1.07	(1.00 to 1.15)	0.056

**lung**	10163	42415	1998 (19.7)	7621 (18.0)	1.16	(1.09 to 1.23)	1.07	(0.99 to 1.16)	0.095

**blood**	7185	29162	973 (13.5)	4339 (14.9)	0.91	(0.84 to 0.99)	0.78	(0.71 to 0.86)	< 0.001

**bladder**	4227	17559	856 (20.3)	3125 (17.8)	1.23	(1.12 to 1.34)	1.15	(1.03 to 1.29)	0.012

**skin**	3249	13115	433 (13.3)	1675 (12.8)	1.12	(0.99 to 1.26)	1.08	(0.93 to 1.26)	0.292

**oesophagus**	3159	13041	496 (15.7)	2106 (16.1)	0.97	(0.87 to 1.09)	0.88	(0.77 to 1.01)	0.072

**pancreas**	2110	8762	365 (17.3)	1397 (15.9)	1.15	(1.01 to 1.32)	0.96	(0.82 to 1.14)	0.671

**stomach**	1992	8279	322 (16.2)	1363 (16.5)	1.00	(0.87 to 1.16)	0.86	(0.72 to 1.02)	0.078

**All cancers**	**88125**	**362254**	**13621 (15.5)**	**54606 (15.1)**	**1.07**	**(1.05 to 1.09)**	**1.01**	**(0.99 to 1.04)**	**0.280**

^# ^Adjusted for Townsend quintile, body mass index, smoking status, myocardial infarction, coronary heart disease, diabetes, hypertension, stroke, rheumatoid arthritis, use of NSAIDs, Cox2-inhibitors, aspirin

† Also adjusted for family history of breast cancer, use of oral contraceptives, hormone-replace therapy

‡ Also adjusted for colitis and Crohn's disease

**Table 3:** Cumulative duration of statin use in cases and controls in 13 to 72 months prior to the index date by cancer site (in cases and matched controls with at least 6 years of medical records)

	Less than 12 months		13 to 24 months		25 to 48 months		49 months and more		
**cancer**	**Cases/Controls**	**Adjusted Odds ratio (95%CI) **^#^	**Cases/Controls**	**Adjusted Odds ratio (95%CI) **^#^	**Cases/Controls**	**Adjusted Odds ratio (95%CI) **^#^	**Cases/Controls**	**Adjusted Odds ratio (95%CI) **^#^	P-* value

breast†	433/1811	1.01 (0.90 to 1.13)	289/1292	0.93 (0.81 to 1.07)	430/1685	1.07 (0.95 to 1.21)	329/1439	0.95 (0.83 to 1.09)	0.719

prostate	668/2784	1.05 (0.95 to 1.15)	560/2187	1.14 (1.03 to 1.26)	796/3295	1.09 (0.99 to 1.19)	750/3242	1.05 (0.95 to 1.16)	0.084

colorectal‡	525/2038	1.05 (0.95 to 1.17)	400/1595	1.04 (0.92 to 1.17)	539/2230	1.02 (0.92 to 1.14)	536/1907	1.23 (1.10 to 1.38)	0.002

lung	485/1857	1.02 (0.90 to 1.15)	406/1478	1.11 (0.97 to 1.27)	549/2233	1.01 (0.90 to 1.14)	558/2053	1.18 (1.05 to 1.34)	0.013

blood	255/1082	0.84 (0.72 to 0.98)	201/860	0.81 (0.68 to 0.96)	277/1307	0.73 (0.63 to 0.84)	240/1090	0.76 (0.65 to 0.89)	< 0.001

bladder	209/785	1.13 (0.95 to 1.34)	174/611	1.18 (0.98 to 1.42)	240/952	1.06 (0.90 to 1.25)	233/777	1.29 (1.08 to 1.54)	0.014

skin	120/422	1.19 (0.95 to 1.49)	61/347	0.74 (0.55 to 0.99)	141/474	1.23 (0.99 to 1.54)	111/432	1.08 (0.84 to 1.39)	0.373

oesophagus	126/571	0.82 (0.67 to 1.02)	97/394	0.91 (0.71 to 1.17)	128/601	0.82 (0.66 to 1.02)	145/540	1.04 (0.83 to 1.30)	0.705

pancreas	87/367	0.85 (0.66 to 1.10)	73/269	1.02 (0.76 to 1.36)	113/390	1.09 (0.86 to 1.40)	92/371	0.97 (0.74 to 1.28)	0.521

stomach	76/317	0.87 (0.66 to 1.15)	69/271	0.90 (0.67 to 1.20)	94/404	0.86 (0.67 to 1.11)	83/371	0.85 (0.64 to 1.12)	0.167

**All cancers**	**3467/13935**	**1.01 (0.97 to 1.05)**	**2752/10855**	**1.02 (0.98 to 1.07)**	**3868/15708**	**1.00 (0.96 to 1.04)**	**3534/14108**	**1.04 (1.00 to 1.09)**	**0.057**

^# ^Adjusted for Townsend quintile, body mass index, smoking status, myocardial infarction, coronary heart disease, diabetes, hypertension, stroke, rheumatoid arthritis, use of NSAIDs, Cox2-inhibitors, aspirin

† Also adjusted for family history of breast cancer, benign breast disease, use of oral contraceptives, hormone-replace therapy

‡ Also adjusted for colitis and Crohn's disease

* Trend test based on number of months prescribed

**Table 4:** Types of statins in cases and controls in 13 to 72 months prior to the index date (in cases and matched controls with at least 6 years of medical records)

	Atorvastatin			Pravastatin			Simvastatin		
**cancer**	**Cases/Controls**	**Adjusted Odds ratio (95%CI) **^#^	**P-value**	**Cases/Controls**	**Adjusted Odds ratio (95%CI) **^#^	**P-value**	**Cases/Controls**	**Adjusted Odds ratio (95%CI) **^#^	**P-value**

breast†	596/2574	0.96 (0.86 to 1.06)	0.387	152/630	1.02 (0.85 to 1.23)	0.835	871/3720	0.98 (0.89 to 1.07)	0.584

prostate	1023/4398	0.99 (0.91 to 1.07)	0.781	314/1182	1.15 (1.00 to 1.31)	0.046	1668/6924	1.05 (0.99 to 1.13)	0.117

colorectal‡	826/2934	1.17 (1.07 to 1.28)	0.001	212/786	1.09 (0.93 to 1.28)	0.289	1152/4783	0.96 (0.88 to 1.04)	0.273

lung	786/2912	1.07 (0.97 to 1.18)	0.179	195/837	0.93 (0.78 to 1.11)	0.435	1205/4588	1.06 (0.97 to 1.15)	0.202

blood	381/1653	0.87 (0.77 to 0.99)	0.041	95/452	0.84 (0.66 to 1.06)	0.138	579/2592	0.82 (0.73 to 0.91)	< 0.001

bladder	353/1212	1.19 (1.03 to 1.37)	0.015	87/309	1.08 (0.84 to 1.40)	0.544	513/1893	1.10 (0.97 to 1.25)	0.119

skin	168/655	1.03 (0.84 to 1.25)	0.805	43/178	0.91 (0.64 to 1.30)	0.609	259/998	1.06 (0.90 to 1.26)	0.483

oesophagus	197/846	0.88 (0.73 to 1.05)	0.159	62/226	1.05 (0.78 to 1.42)	0.740	298/1222	0.94 (0.80 to 1.09)	0.403

pancreas	143/557	0.92 (0.74 to 1.14)	0.439	37/152	0.92 (0.62 to 1.35)	0.667	224/852	0.99 (0.83 to 1.20)	0.952

stomach	123/500	0.94 (0.75 to 1.18)	0.604	41/149	1.03 (0.71 to 1.50)	0.880	186/819	0.85 (0.70 to 1.03)	0.106

**All cancers**	**5357/21253**	**1.01 (0.98 to 1.05)**	**0.461**	**1442/5680**	**1.02 (0.96 to 1.09)**	**0.488**	**8102/32769**	**1.00 (0.97 to 1.03)**	**0.844**

^# ^Adjusted for Townsend quintile, body mass index, smoking status, myocardial infarction, coronary heart disease, diabetes, hypertension, stroke, rheumatoid arthritis, use of NSAIDs, Cox2-inhibitors, aspirin

† Also adjusted for family history of breast cancer, benign breast disease, use of oral contraceptives, hormone-replace therapy

‡ Also adjusted for colitis and Crohn's disease

**Table 5:** Cumulative duration of statin use in cases and controls in 13 to 120 months prior to the index date by cancer site in cases and controls with 10 or more years of recorded data

	Less than 12 months		13 to 24 months		25 to 48 months		49 to 72 months		73 months and more		
**cancer**	**Cases/Controls**	**Adjusted Odds ratio (95%CI) **^#^	**Cases/Controls**	**Adjusted Odds ratio (95%CI) **^#^	**Cases/Controls**	**Adjusted Odds ratio (95%CI) **^#^	**Cases/Controls**	**Adjusted Odds ratio (95%CI) **^#^	**Cases/Controls**	**Adjusted Odds ratio (95%CI)**^#^	P-* value

**breast**†	363/1338	1 (0.86 to 1.16)	254/962	0.96 (0.81 to 1.15)	346/1208	1.06 (0.90 to 1.25)	169/634	0.93 (0.75 to 1.15)	131/542	0.85 (0.67 to 1.08)	0.220

**prostate**	545/2119	0.99 (0.87 to 1.12)	489/1609	1.19 (1.04 to 1.36)	641/2424	1.06 (0.94 to 1.19)	369/1394	1.07 (0.93 to 1.24)	320/1234	1.12 (0.96 to 1.32)	0.173

**colorectal**‡	446/1527	1.06 (0.92 to 1.23)	327/1224	1.03 (0.88 to 1.21)	429/1625	0.97 (0.84 to 1.13)	285/902	1.20 (1.01 to 1.43)	213/711	1.21 (0.99 to 1.48)	0.069

**lung**	419/1375	1.06 (0.91 to 1.25)	342/1064	1.21 (1.01 to 1.43)	458/1586	1.05 (0.89 to 1.23)	259/950	1.00 (0.82 to 1.21)	235/734	1.17 (0.95 to 1.45)	0.240

**blood**	202/821	0.72 (0.59 to 0.88)	160/616	0.8 (0.64 to 1.00)	233/966	0.74 (0.61 to 0.89)	133/468	0.87 (0.68 to 1.11)	90/428	0.55 (0.41 to 0.73)	< 0.001

**bladder**	172/565	1.14 (0.91 to 1.43)	137/456	1.19 (0.93 to 1.53)	190/681	1.01 (0.81 to 1.26)	117/360	1.19 (0.91 to 1.57)	94/280	1.37 (1.02 to 1.86)	0.062

**skin**	110/312	1.41 (1.04 to 1.89)	56/270	0.79 (0.55 to 1.13)	112/349	1.21 (0.89 to 1.64)	50/176	0.87 (0.56 to 1.36)	56/185	1.04 (0.70 to 1.55)	0.626

**oesophagus**	103/409	0.9 (0.67 to 1.20)	75/272	1.01 (0.72 to 1.42)	105/445	0.81 (0.61 to 1.09)	68/248	1.09 (0.77 to 1.55)	59/185	1.03 (0.70 to 1.52)	0.870

**pancreas**	76/277	0.89 (0.63 to 1.24)	61/196	1.22 (0.84 to 1.78)	86/286	0.99 (0.71 to 1.38)	50/172	1.04 (0.69 to 1.56)	40/148	0.85 (0.53 to 1.37)	0.475

**stomach**	58/232	0.84 (0.58 to 1.22)	60/206	0.82 (0.56 to 1.19)	77/301	0.86 (0.61 to 1.21)	35/150	0.56 (0.35 to 0.90)	38/165	0.63 (0.39 to 1.00)	0.008

***All cancers***	**2890/10413**	**0.99 (0.94 to 1.05)**	**2303/8005**	**1.05 (0.99 to 1.11)**	**3107/11437**	**0.98 (0.93 to 1.03)**	**1776/6319**	**1.01 (0.95 to 1.08)**	**1462/5259**	**1.02 (0.95 to 1.10)**	**0.958**

^# ^Adjusted for Townsend quintile, body mass index, smoking status, myocardial infarction, coronary heart disease, diabetes, hypertension, stroke, rheumatoid arthritis, use of NSAIDs, Cox2-inhibitors, aspirin

† Also adjusted for family history of breast cancer, benign breast disease, use of oral contraceptives, hormone-replace therapy

‡ Also adjusted for colitis and Crohn's disease

* Trend test based on number of months prescribed

#### 4.2.1 Cancer of any site

The analysis for overall risk of cancer (at any site) did not show a significant association with any statin use (Table 2). Patients with a cumulative prescription duration of more than one year had a similar risk of cancer of any site compared with patients with no statin prescriptions (adjusted odds ratio (AOR) 1.02, 95%CI 0.99 to 1.05). Analyses of trends for duration of use (Table 3) and dosage, as well as analysis of use of individual statins (Table 4), did not show any effect of statins on overall risk of cancer.

#### 4.2.2 Colorectal cancer

There was no overall increase of colorectal cancer risk in statin users (AOR 1.07, 95%CI 1.00 to 1.15, P = 0.056), with a slight association for patients with prescriptions for more than a year (AOR = 1.09, 95%CI 1.01 to 1.18, P = 0.036), which was not however statistically significant at P < 0.01. Further analysis showed a significant association with duration of use of statins (P_trend_= 0.001), with a 23% increased risk for 49 to 60 months of use of (AOR 1.23, 95%CI 1.10 to 1.38) compared with no use. The analysis of the median prescribed dose of statins revealed a significant association with an 18% increased risk on high dose of statin (AOR 1.18, 95%CI 1.07 to 1.31, P = 0.001).

Analyses of individual statins showed an association between colorectal cancer and atorvastatin (P_trend_= 0.001), with an increased risk of colorectal cancer associated with atorvastatin use of 4 or more years (AOR 1.51, 95%CI 1.24 to 1.83).

The risk of colorectal cancer was not significantly increased for patients who stopped taking statins more than 2 years before the index date (AOR 1.03, 95%CI 0.81 to 1.31).

The increased risk of colorectal cancer associated with longer duration of statin use found in patients with at least 6 years of medical records was not supported by the trend test of months on medication in patients with at least 10 years of medical records (p = 0.069).

#### 4.2.3 Bladder cancer

For bladder cancer, there was a borderline 15% increased risk of cancer associated with any use of statins (P = 0.012) and a 16% increased risk associated with more than one year's use (P = 0.018), but these were not statistically significant. For patients with more than 48 months of statin use, risk of bladder cancer was 29% higher (AOR, 1.29, 95% 1.08 to 1.54, P = 0.006) but the trend test for duration was not statistically significant (P_trend _= 0.014). No particular type of statin was significantly associated with an increased risk. The risk of bladder cancer was not significantly increased in patients who stopped taking statins more than 2 years before the index date (AOR 0.94, 95%CI 0.62 to 1.40).

The additional analysis restricted to patients with at least 10 years of medical records showed similar results, but these were not statistically significant.

#### 4.2.4 Lung cancer

Although the unadjusted risk of lung cancer appeared to be significantly higher in statin users (unadjusted odds ratio (UOR) 1.16, 95%CI 1.09 to 1.23, P < 0.001), after adjusting for cardiovascular disease the association became much weaker (OR 1.07, 95%CI 1.00 to 1.14, P = 0.067) and did not noticeably change after further adjusting for other factors.

The unadjusted trend test for months of statin use was significant (P < 0.001) and use of statins for more than 4 years was associated with an increased risk of cancer (UOR 1.22, 95%CI 1.10 to 1.35, P < 0.001). After adjusting for cardiovascular disease and other factors, these associations were also reduced but long-term usage remained significant (AOR 1.18, 95%CI 1.05 to 1.34, P = 0.007).

Analyses repeated on patients with 10 years of medical records did not show any statistically significant effect of statins for either overall or long term use.

#### 4.2.5 Prostate cancer

Although the analysis demonstrated an 8% increased risk of prostate cancer for overall statin user and a 9% increased risk for patients with prescriptions covering more than a year, these associations were not statistically significant (P = 0.016 and P = 0.011). There were no dose or duration relationships in patients with either 6 years or 10 years of medical records.

#### 4.2.6 Haematological malignancies

There was a 22% reduced blood cancer risk for overall statin use (AOR 0.78, 95%CI 0.71 to 0.86, P < 0.001) and a 24% reduction for patients with statin prescriptions of more than a year (AOR 0.76, 95%CI 0.68 to 0.85), with a significant trend for duration of use (P_trend _< .001). No differential effects were found for particular types of statin. Patients who stopped taking statins for more than 2 years had the same risk of cancer as non-statin users (AOR 0.90, 95%CI 0.67 to 1.23).

Although lymphoma, myeloma and leukaemia were similarly associated with overall use of statins and use for more an year, only leukaemia had associations with duration and dose with significant trend tests (P_trend _= 0.002 and P_trend _< 0.001), a 26% risk reduction (AOR 0.74, 95%CI 0.62 to 0.87, P = 0.001) with at least two years of statin prescriptions, and a 25% risk reduction on high dose (AOR 0.75, 95%CI 0.61 to 0.92, P = 0.006).

#### 4.2.7 Other cancers

There were no significant associations with statin use for any other cancers.

#### 4.2.8 Sensitivity analyses

The sensitivity analyses treating missing values for smoking, and body mass index as separate categories produced very similar results. The complete case analyses resulted in very similar odds ratios, but the confidence intervals were wider due to the reduced number of observations (results available from the authors).

## 5 Discussion

In this large population-based case control study to determine the risk of common cancers associated with use of statins, we confirmed that use of statins does not affect the overall risk of cancer. We did find some evidence of an increased risk of colorectal cancer in patients using statins for 4 or more years or with a high statin dose. We also found an increased risk of bladder cancer and lung cancer in patients prescribed statins for 4 or more years. Conversely, we found a reduced risk of haematological malignancies in statin users.

There are a large number of studies devoted to statins and cancer risk summarised in meta-analyses[6-8] which did not show an adverse or protective effect of statins on the overall incidence of cancer. However, the categorisation of 'any cancer' is not a specific enough endpoint of study as it covers a range of diseases, each with a different aetiology and course of development.

Colorectal cancer, as one of the most common cancers, has been studied extensively but only eight epidemiological studies looked at the effect of long-term statin use (at least 4 years). Four of them[38-41] had odds ratios greater than unity (from 1.00 to 1.15) and four of them[22,42-44] reported odds ratios less than unity (from 0.71 to 0.83), but none of these findings reached statistically significant levels even at the 5% level. The effect of dose in our study might, however, be a replication of the effect of cumulative use because a high dose was more likely to be prescribed for patients who had been on statins for substantial period of time.

The other two most common cancers, breast and prostate, also account for a number of studies but there has been no definite outcome in associating any of these with use of statins and our null results are consistent with this. Studies for prostate cancer have been aggregated into a meta-analysis [45], which did not find any significant association with overall risk of prostate cancer and another meta-analysis[46] looking at breast cancer studies also failed to demonstrate a protective or adverse effect of statins.

For bladder cancer, results of a meta-analysis considering 5 studies showed an increased, but not significant, association between stain use and cancer risk[7]. There have been very few studies investigating the long-term effect of statin use on bladder cancer. One study[39] showed an increased risk for more than 5 years of statin use, which is consistent with our findings, but another very recent one found no significant association for current use of statins for more than 5 years [22]. Both studies, however, were much smaller.

Our findings of a significant increase in unadjusted lung cancer risk for statin use and for long-term use were both significantly decreased by adjusting for cardiovascular disease, but after adjusting for all factors, long-term use still showed a significant association with increased lung cancer risk. There is no causal link between cardiovascular disease and lung cancer but there is a strong association of both conditions with smoking. The finding about possible increased risk from long-term use is consistent with the results of two other studies[22,39], although their findings were not significant.

The decreased risk of haematological malignancies could be explained by reverse causality, as patients with such diagnoses are more likely to have lower lipid levels[47] although we did restrict our statin exposure to prescriptions at least 12 months before diagnosis. The effect of statins on leukaemia has been studied *in vitro *and there is evidence that statins might suppress the growth of promyelocitic[48] and lymphocytic[49] leukemic cells. However, no epidemiological studies have provided significant evidence of any statin effect on incidence of leukaemia.

Our study has several strengths. It is substantially larger and has greater statistical power than any previous study. This has allowed us to perform the analyses separately for different cancers within the same population. We had a substantial number of patients with at least 10 years of records, which also allowed us to examine long-term statin use. The study is based on computer-recorded prescribing and morbidity data collected prospectively. The study was not subject to response bias or recall bias as the exposure data were recorded before the date of diagnosis or pseudo-diagnosis. Any bias from misclassification is likely to be small because the level of accuracy and completeness of medical records in general practices has been shown to be high [50].

Matching the controls on sex, age, practice and calendar year removed confounding by these factors. Any bias from misclassification of statin use is likely to be minimal as more than 99% of all general practitioners' repeat prescriptions are recorded on computer[51]. We minimised the possibility of misleading data from the effects of undiagnosed cancer in new medical records by excluding prescriptions, diagnoses of co-morbidities and records of body mass index made within the 12 months prior to the date of the diagnosis or pseudo-diagnosis of cancer.

Our study has some limitations. Information on certain risk factors for cancer, such as level of physical activity, alcohol use, and diet, and information on cancer screening tests (mammography, prostate-specific antigen test and colonoscopy) were not reliably recorded on the database and not included in the analysis so there may be some residual confounding. Although we adjusted the risk of cancer for possible effects of smoking, obesity, deprivation, co-morbidities and the use of other medications, residual confounding may also result from misclassification of those variables. Values of body mass index or smoking status, were missing for about 22% of cases and 25% of controls, so we substituted missing values using multiple imputation. We did not include blood test results in the analysis, in particular high-density lipoproteins and total-serum cholesterol, because they were not consistently recorded on the data base and would be more likely to be recorded in statin users.

Although our data contain detailed information on drug prescriptions, this may not reflect actual use. However there is no reason to think that any non-adherence would systematically differ between cases and controls.

Another possible source of misclassification arises from a statin (simvastatin 10 mg) having become available over the counter in May 2004 in the UK, which would affect mostly younger people who are not entitled to free prescriptions[52] and only a small part of the study period. However, among statin users 81.4% of cases and 82.4% of controls were aged 65 years or older and therefore entitled to free prescribed medications. Analyses repeated on this group of patients obtained similar results, which suggests that any misclassification of use of medication because of over-the-counter purchase is not an explanation for our findings.

## 6 Conclusion

In summary, we have conducted a large population-based case-control study that examined the effect of statins on the risk of cancer and found that there is no effect from prolonged use of statins on overall risk of cancer, but that prolonged use of statins may be associated with an increased risk of colorectal cancer, bladder cancer and lung cancer and a decreased risk of haematological malignancies.

## 7 Approvals

This project has been approved by the QRESEARCH scientific board and notified to the Trent Multi Centre Research Ethics Committee.

## 8 Funding

There was no external funding for the study.

## 11 Competing interests

JHC is codirector of QResearch which is a not-for-profit partnership between the University of Nottingham and EMIS.

## 9 Authors' contributions

YV contributed to the study design, data extraction, data manipulation, data analysis, interpretation and drafting of the paper. JHC had the original idea for this study and extracted the data, contributed to the interpretation and drafting of the paper. CC contributed to the development of the idea, interpretation and drafting of the paper. YV is the guarantor of the study. All authors read and approved the final manuscript.

## Pre-publication history

The pre-publication history for this paper can be accessed here:

http://www.biomedcentral.com/1471-2407/11/409/prepub
